# A distinct antigen presentation pathway drives potent T cell immunity in lipid nanoparticle–based mRNA vaccines

**DOI:** 10.1126/sciadv.aec7827

**Published:** 2026-07-17

**Authors:** Ryunosuke Muro, Suqi Wang, Taku Ito-Kureha, Hung Hiep Huynh, Kouji Kobiyama, Takuya Sugita, Kazuo Okamoto, Kenta Nakano, Tadashi Okamura, Masato Kubo, Ken J. Ishii, Takeshi Nitta, Hiroshi Takayanagi

**Affiliations:** ^1^ Department of Immunology, Graduate School of Medicine and Faculty of Medicine, The University of Tokyo, Tokyo, Japan.; ^2^ Division of Molecular Pathology, Research Institute for Biomedical Sciences, Tokyo University of Science, Chiba, Japan.; ^3^ Division of Vaccine Science, Department of Microbiology and Immunology, The Institute of Medical Science, The University of Tokyo, Tokyo, Japan.; ^4^ International Vaccine Design Center, The Institute of Medical Science, The University of Tokyo, Tokyo, Japan.; ^5^ Division of Rheumatology, Department of Medicine, University of California San Diego, La Jolla, CA, United States.; ^6^ Division of Immune Environment Dynamics, Cancer Research Institute, Kanazawa University, Kanazawa, Japan.; ^7^ Immune Network Research Unit, Institute for Frontier Science Initiative InFiniti, Kanazawa University, Kanazawa, Japan.; ^8^ Department of Laboratory Animal Medicine, National Institute of Global Health and Medicine, Japan Institute for Health and Security (JIHS), Tokyo, Japan.; ^9^ Kyoto University Immunomonitoring Center, Liaison Office, Kyoto University, Kyoto, Japan.; ^10^ Department of Immunology, Graduate School of Medicine, Kyoto University, Kyoto, Japan.

## Abstract

Lipid nanoparticle–encapsulated mRNA (mRNA-LNP) vaccines trigger the potent differentiation of antigen-specific cytotoxic CD8 T cells in addition to antibody production. Despite its high immunogenicity, the cellular mechanisms by which mRNA-LNP induces such unusual immune responses remain largely unclear. Here, we show that mRNA-LNP induces potent and long-lasting CD8 T cell expansion through an antigen presentation mechanism that differs from that of conventional adjuvants. In mice immunized with mRNA-LNP, the number of antigen-specific CD8 T cells was one order of magnitude higher than that induced by combining antigen proteins with immunostimulants such as lipopolysaccharide or polyinosinic:polycytidinic acid. Intramuscularly administered mRNA-LNPs were mainly taken up by migratory type 2 conventional dendritic cells in draining lymph nodes, resulting in notably strong and persistent antigen presentation through major histocompatibility complex class I. Furthermore, CD8 T cell induction by mRNA-LNP required migratory dendritic cells but not the traditional cross-presentation pathway that is otherwise essential for antiviral and antitumor immunity. Thus, the mRNA-LNP formulation exerts unconventional immune responses through a different antigen-presentation pathway from conventional component vaccines.

## Teaser

mRNA-LNP vaccines elicit potent CD8 T cell responses through antigen presentation pathways distinct from conventional vaccines.

## INTRODUCTION

Antigen presentation is an important process that bridges the gap between innate and antigen-specific acquired immunity, playing a vital role in immune responses to pathogens and vaccines. Exogeneous antigens endocytosed by antigen-presenting cells are degraded in lysosomes to form peptides, which are then presented by major histocompatibility complex (MHC) class II proteins to activate CD4 T cells, leading to the production of antigen-specific antibodies. Classical dendritic cells (cDCs) are efficient antigen-presenting cells, and comprise two functionally distinct subsets: cDC1 and cDC2 (*1*, *2*). In particular, the cDC1 subset plays a crucial role in the immune response by uniquely presenting exogenous antigens to cytotoxic CD8 T cells via MHC class I, thereby priming them to combat viral infections. This process, known as cross-presentation, plays a major role in initiating immune responses against pathogens (*3*).

Messenger RNA (mRNA)–lipid nanoparticle (LNP) products have been used as vaccines against COVID-19 in many countries (*4*–*6*). The versatility of mRNA vaccines lies in their ability to encode virtually any antigen by designing the corresponding mRNA sequence, enabling them to target a wide range of pathogens or tumors. Preclinical and clinical studies have examined the immunogenicity of mRNA-LNP, including antibody and T cell responses (*7*, *8*). The modification of ribonucleotides in mRNA is a key strategy for suppressing interferon (IFN) responses and enhancing the production of large amounts of antigen protein. In addition, the ionizable lipid component of LNPs can function as an adjuvant, leading to inflammation and activation of acquired immunity (*9*). However, the full mechanism by which mRNA-LNP induces a strong cytotoxic T cell response is still unclear, and further research is needed (*10*, *11*).

mRNA-LNP vaccines induce different types of immune responses compared to conventional component vaccines. First, mRNA-LNP is excellent at inducing cytotoxic CD8 T cells. Clinical trials have demonstrated a significant increase in antigen-specific CD8 T cells following mRNA-LNP administration, even at a dose insufficient to stimulate antibody production (*7*, *12*). In mouse studies, it has been suggested that the induction of CD8 T cells by mRNA-LNP is mediated through the cross-presentation of mRNA-encoded antigen by cDC1 cells (*8*); however, another study showed that cDC1 cells are not required (*13*), representing a discrepancy in the understanding of the mechanism underlying mRNA-LNP-induced T cell priming. Second, IgG4 antibodies are markedly increased in people who have been vaccinated with mRNA-LNP (*14*–*16*). IgG4 is an immunosuppressive class of antibody, and its production mechanism and role in vaccine effectiveness are unknown. Third, mRNA-LNP strongly induces the differentiation of IFNγ-producing helper T (Th) cells compared to other Th cell lineages (*12*). These are unique immune responses observed in mRNA-LNP, but the details of the mechanism have not been elucidated.

The aim of this study was to determine the mechanism by which mRNA-LNPs induce immune responses utilizing mice administered mRNA-LNP encoding model antigens. Immunization with mRNA-LNP encoding ovalbumin (OVA) preferentially induced the differentiation of Th1 and the production of OVA-specific IgG2b and IgG2c rather than IgG1 antibodies. In addition, OVA mRNA-LNP induced the robust expansion of OVA-specific CD8 T cells compared to immunization with OVA protein and conventional adjuvants. Results with green fluorescent protein (GFP)–encoding mRNA showed that intramuscularly administered LNPs were mainly taken up by migratory cDC2 cells in draining lymph nodes (dLNs), and induced antigen presentation much more strongly than conventional immunization methods using recombinant proteins and adjuvants. Furthermore, antigen presentation by mRNA-LNP required migratory DCs (mDCs) but not cross-presentation for inducing CD8 T cell responses. The results of this study provide new insights into the mechanism of mRNA-LNP-induced T cell immunity, contributing to the development of vaccines for serious viral infections.

## RESULTS

### mRNA-LNP induces the high production of IgG2b/IgG2c antibodies

**Fig. 1.**
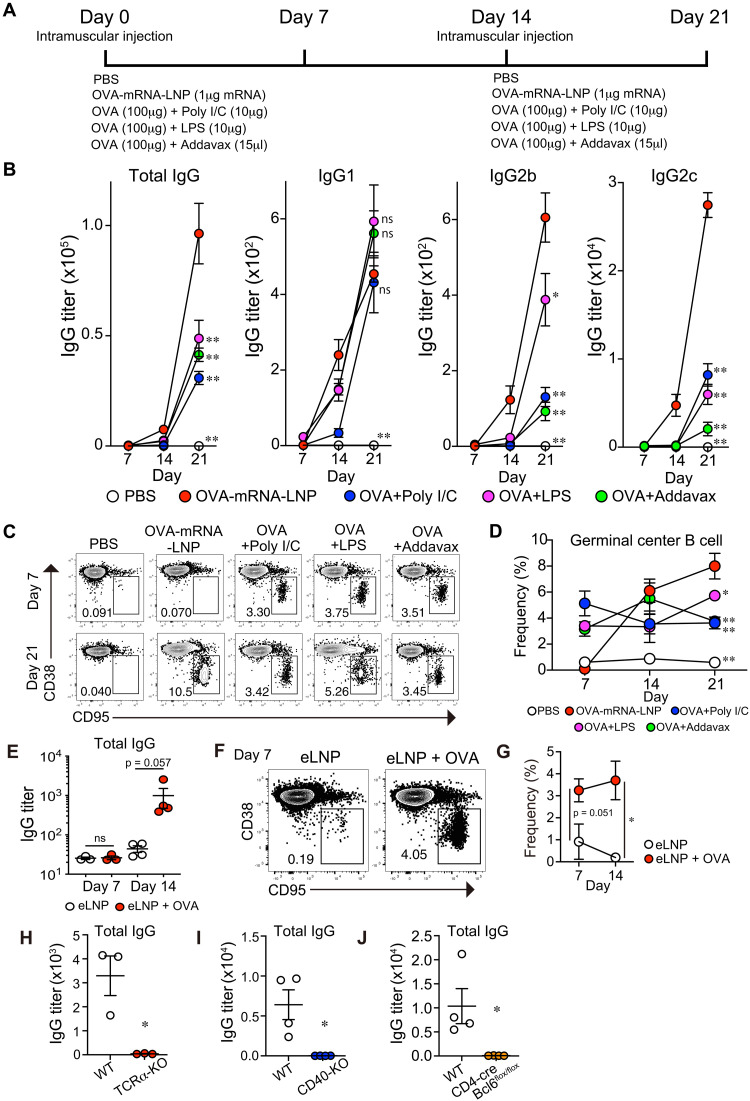
Skewed IgG subclasses produced by mRNA-LNP. (**A**) Experimental schedule of immunization. OVA-mRNA-LNP or protein combined with stimulants (poly I/C, LPS, or AddaVax) was administered intramuscularly. A booster immunization was performed via the same route 14 days later. (**B**) Quantification of OVA-specific IgG antibodies in serum using enzyme-linked immunosorbent assay (ELISA). PBS, *n* = 3–7; OVA-mRNA-LNP, *n* = 4–10; OVA+ poly I/C, *n* = 5; OVA + LPS, *n* = 5–8; OVA + AddaVax, *n* = 5. (**C** and **D**) Flow cytometry analysis of germinal center B cells (CD19^+^ CD95^+^ CD38^lo^) in the popliteal LNs on days 7 and 21 (C). The graph represents the change in the frequency of GC B cells over time (D). PBS, *n* = 3–5; OVA-mRNA-LNP, *n* = 4–7; OVA + poly I/C, *n* = 5; OVA + LPS, *n* = 5; OVA + AddaVax, *n* = 5. (**E**) OVA-specific IgG antibody titer in the serum of WT mice immunized with either eLNP or eLNP + OVA (*n* = 3–4 per group). (**F** and **G**) Representative flow cytometry plots showing the formation of germinal center B cells in the popliteal LNs of the indicated mice (F). The graph shows the frequency of germinal center B cells on days 7 and 14 (*n* = 3–4 per group) (G). (**H** to **J**) Serum OVA-specific IgG levels were evaluated by ELISA on day 14 in wild-type (WT; *n* = 3–4), TCR-α knockout (KO) mice [(H), *n* = 3], CD40 KO mice [(I), *n* = 4], and CD4cre Bcl6^flox/flox^ mice [(J), *n* = 4]. Statistical significance was determined by one-way analysis of variance (ANOVA) followed by Dunnett’s multiple comparisons test for (B) and (D), and two-tailed Student’s *t* test for (E) and (G) to (J); *P < 0.05, **P < 0.01. ns, not significant.

To examine the immune responses induced by the mRNA-LNP formulation, we immunized C57BL/6 mice with OVA-encoding mRNA encapsulated in LNPs of the Pfizer/BioNTech type composition. These OVA mRNA-LNPs (1 μg RNA equivalent) were administered via intramuscular injection according to the schedule shown in Fig. 1A. As a comparison for the immune responses, OVA protein (100 μg) with conventional immunostimulants including *Escherichia coli* lipopolysaccharide (LPS), polyinosinic:polycytidinic acid (poly I/C), or AddaVax were also used.

First, we measured the antibody production induced by vaccination. OVA-specific IgG antibodies in serum were detectable on day 14, and continued to increase until day 21 (7 days after the booster) (Fig. 1B). Mice administered OVA mRNA-LNP produced significantly higher levels of OVA-specific IgG antibodies on days 14 and 21 than mice treated with OVA proteins and adjuvants. Among the OVA-specific IgG antibodies, the levels of IgG1 were similar between mRNA-LNP and OVA protein + adjuvants, whereas the levels of IgG2b and IgG2c were significantly higher in OVA mRNA-LNP-treated mice than in other groups. The most significantly induced IgG antibody by mRNA-LNP was IgG2c, with levels induced 3- to 15-fold higher compared to other groups. Therefore, the high production of IgG antibodies by mRNA-LNP was due to an increase in IgG2c and not IgG1.

Next, germinal center B cells in dLNs, which play an important role in antibody production, were examined. On day 7, CD95^hi^CD38^lo^ germinal center B cells were induced in mice treated with OVA protein + conventional adjuvants (poly I/C, LPS, or AddaVax), but not in mice treated with OVA mRNA-LNP (Fig. 1, C and D and fig. S1A). After day 14, however, the frequency of germinal center B cells was higher in mice treated with OVA mRNA-LNP compared to other groups. These germinal center B cells contained OVA-specific cells stained with fluorochrome-conjugated OVA protein, and their numbers markedly increased following booster immunization (fig. S1, B and C).

It has been reported that the ionizable lipids in LNPs can act as adjuvants that enhance immune response (*9*). Indeed, mice immunized with OVA protein and empty LNPs (eLNPs) produced OVA-specific IgG (Fig. 1E). However, in mice administered OVA protein and eLNP, germinal center B cells were detected on day 7, indicating that the kinetics of the B cell responses differed from that induced by OVA-mRNA-LNP (Fig. 1, F and G). These findings suggest that the delayed germinal center B cell formation observed with OVA mRNA-LNP cannot be explained by the action of LNP itself.

We further investigated memory B cell responses induced by OVA mRNA-LNP. Mice were immunized with either OVA mRNA-LNP or OVA protein + poly I/C, rested for 8 weeks, and subsequently challenged with OVA protein (100 μg) alone to assess recall responses (fig. S2A). No significant differences in IgG1 or IgG2b recall responses were observed between the two groups (fig. S2B). In contrast, mice primed with OVA mRNA-LNP exhibited robust IgG2c induction upon recall, whereas such a pronounced response was not observed in the OVA protein + poly I/C group. These results indicate that the mRNA-LNP formulation results in quantitatively and qualitatively different B-cell responses than conventional adjuvants in mice.

Antibody production by mRNA-LNP is entirely T cell-dependent, as T cell receptor alpha (TCRα)-deficient mice failed to produce detectable OVA-specific IgG following administration of OVA mRNA-LNP (Fig. 1H). OVA-specific IgG was also undetectable in CD40-deficient mice and T cell-specific Bcl6-deficient (Cd4Cre Bcl6^flox/flox^) mice, indicating that mRNA-LNP-induced antibody production is dependent on the interaction between Th cells and B cells in the germinal centers of LNs (Fig. 1, I and J).

### mRNA-LNP induces Th1 skewing

**Fig. 2.**
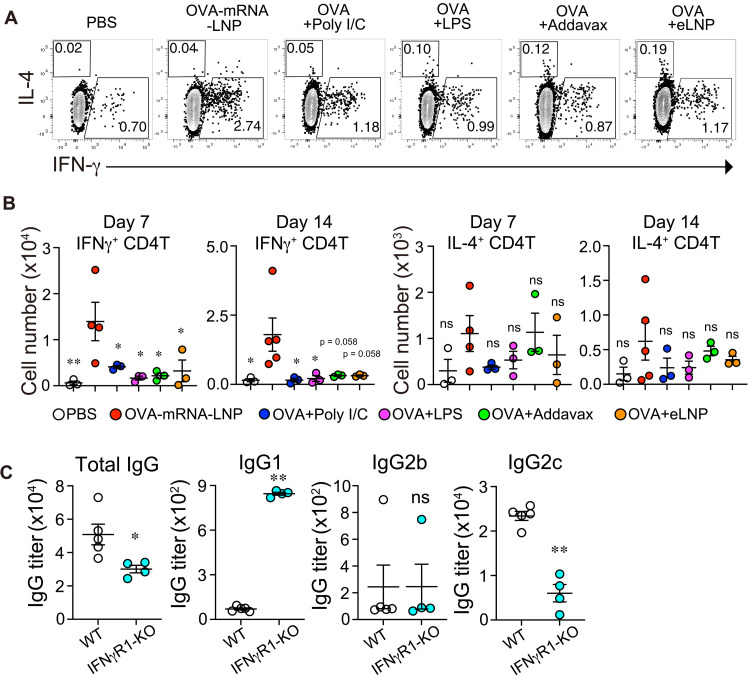
mRNA-LNP promotes Th1 differentiation. (**A** and **B**) Intracellular staining of IFNγ and IL-4 in CD4 T cells from the popliteal LNs after stimulation with phorbol myristate acetate (PMA) and ionomycin. Cells were collected on day 14 from mice immunized with OVA-mRNA-LNP or OVA protein combined with adjuvants (poly I/C, LPS, AddaVax, or eLNP (A). The graph shows the number of IFNγ^+^ or IL-4^+^ CD4 T cells on days 7 and 14 (B). PBS, *n* = 3; OVA-mRNA-LNP, *n* = 4–5; OVA+ poly I/C, *n* = 3; OVA + LPS, *n* = 3; OVA + AddaVax *n* = 3; OVA + eLNP, *n* = 3. (**C**) Serum OVA-specific IgG levels in WT (*n* = 5) and Ifngr1^−/−^ mice (*n* = 4) were determined by ELISA. Statistical significance was determined by one-way ANOVA followed by Dunnett’s multiple comparisons test for (B), and two-tailed Student’s *t* test for (C); *P < 0.05, **P < 0.01. ns, not significant.

The class of antibodies produced by immune responses depends on the subset of Th cells induced. Previous studies have shown that Th1 cells that produce IFNγ are essential for the production of IgG2c, whereas the follicular helper T (Tfh) subset is required for IgG1 production (*17*, *18*). We examined the differentiation potential of Th cells in the dLNs of immunized mice (Fig. 2, A and B). Mice immunized with OVA mRNA-LNP induced greater differentiation of IFNγ-producing Th1 cells than those immunized with OVA protein + conventional adjuvants or eLNPs. There was no difference in the differentiation of IL-4-producing Th2 cells between the two groups. Thus, the mRNA, not the LNP component, in mRNA-LNP formulations promotes Th1-skewed Th cell differentiation.

Furthermore, in mice lacking the IFNγ receptor (*Ifngr1*^−/−^), the amount of OVA-specific IgG2c produced upon OVA mRNA-LNP administration was significantly lower than that in control mice (Fig. 2C). These results indicate that the mRNA-LNP formulation skews Th cell differentiation towards Th1-producing IFNγ, which promotes class switching in B cells and the high production of IgG2c antibodies.

### mRNA-LNP induces the robust expansion of antigen-specific CD8 T cells

**Fig. 3.**
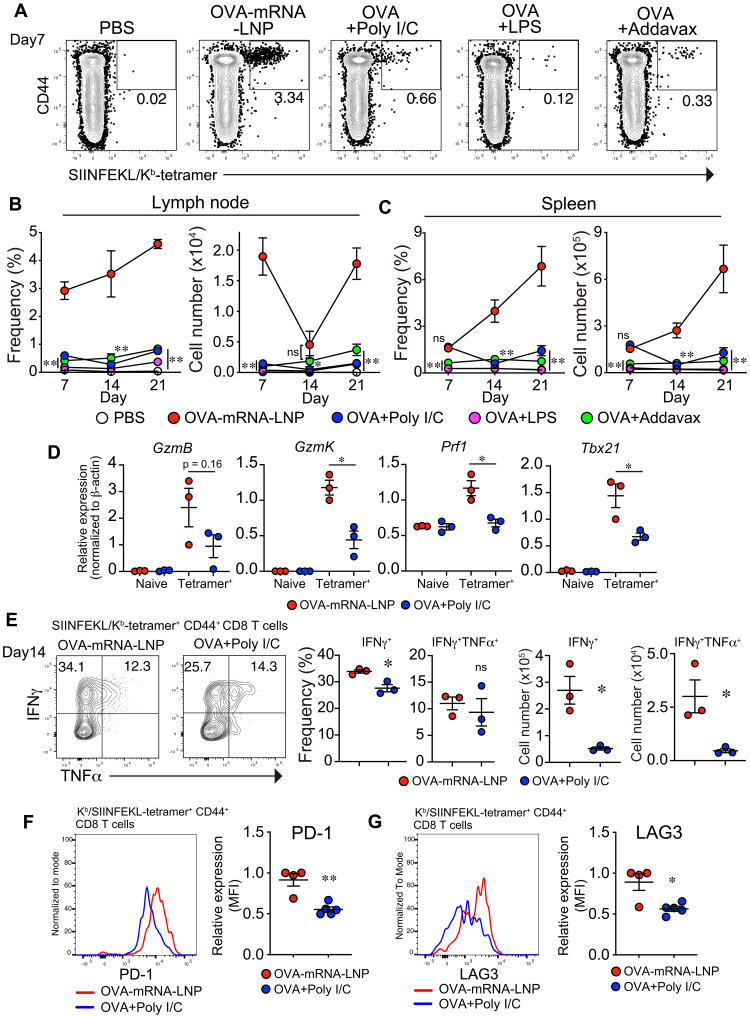
Robust expansion of antigen-specific CD8 T cells by mRNA-LNP. (**A** to **C**) Representative flow cytometry plots showing OVA-specific CD8 T cells, detected by CD44^+^ and SIINFEKL/K^b^-tetramer^+^, in the popliteal LNs of the indicated mice (A). The graphs indicate time-course changes in the number and frequency of OVA-specific CD8 T cells in the LN (B) and spleen (C) over time. PBS, *n* = 3–4; OVA-mRNA-LNP, *n* = 4–7; OVA+ poly I/C, *n* = 5; OVA + LPS, *n* = 5; OVA + AddaVax, *n* = 5. (**D**) mRNA expression levels of *GzmB*, *GzmK*, *Prf1*, and *Tbx21* were determined by qRT-PCR in sorted CD44^lo^ naïve CD8 T cells and CD44^hi^ tetramer^+^ OVA-specific CD8 T cells from the spleens (*n* = 3 per group). (**E**) Expression of IFNγ and TNFα in splenic OVA-specific CD8 T cells was analyzed by flow cytometry on day 14 after immunization. The graphs show the frequency and number of IFNγ single-positive and IFNγ/TNFα double-positive OVA-specific CD8 T cells (*n* = 3 per group). (**F** and **G**) Expression of PD-1 (F) and LAG3 (G) in OVA-specific CD8 T cells was measured by flow cytometry on day 7 after immunization. The mean fluorescent intensity (MFI) of PD-1 and LAG3 is shown as relative expression in the graphs. OVA-mRNA-LNP, *n* = 4; OVA + poly I/C, *n* = 5. Statistical significance was determined by one-way ANOVA followed by Dunnett’s multiple comparisons test for (B) and (C), and two-tailed Student’s *t* test for (D) to (G); *P < 0.05, **P < 0.01. ns, not significant.

mRNA-LNP efficiently induces antigen-specific CD8 T cells (*12*, *19*); however, the quantitative and qualitative differences from component vaccines using conventional adjuvants are unknown. We measured the induction of OVA-specific CD8 T cells in immunized mice, using the OVA peptide/MHC class I (SIINFEKL/K^b^) tetramer. Figure 3A shows the frequency of tetramer^+^ CD44^hi^ effector CD8 T cells in dLNs. Mice treated with OVA mRNA-LNP had 5- to 27-fold more OVA-specific CD8 T cells than the other immunized groups. The frequency of OVA-specific CD8 T cells continued to increase in the spleen as well as in the dLNs in mice treated with OVA-mRNA-LNP (Fig. 3, B and C). On day 21, the number of OVA-specific CD8 T cells in the spleen was 5- to 30-fold higher in OVA-mRNA-LNP-treated mice compared to the OVA protein + adjuvant groups. Conversely, OVA protein + eLNP did not produce such strong and sustained induction of OVA-specific CD8 T cells (fig. S3), indicating that the antigen-coding mRNA encapsulated in LNP was responsible for these effects. In addition, the frequency of OVA-specific CD8 T cells induced by recall response was higher in OVA mRNA-LNP-treated mice than in OVA protein + poly I/C-treated mice, indicating the establishment of long-lasting CD8 T cell memory by mRNA-LNPs (fig. S2C).

Is the route of administration of mRNA-LNP critical for the induction of antigen-specific CD8 T cells? Our results showed no significant differences in the frequency or absolute number of OVA-specific CD8 T cells in the spleen between intramuscular and subcutaneous administration of OVA mRNA-LNP (fig. S4). Furthermore, even when the subcutaneous dose of OVA protein was increased (up to 400 μg), the number of induced CD8 T cells did not reach the levels observed with OVA mRNA-LNP (1 μg) administration. Thus, the potent immune responses induced by OVA mRNA-LNP stems not simply from antigen dose, but rather from the intrinsic properties of the mRNA-LNP formulation itself.

We further examined the expression of cytotoxic effector molecules in the induced CD8 T cells. Compared with CD44^lo^ naïve CD8 T cells, sorted OVA-specific CD8 T cells displayed markedly elevated mRNA expression of Granzyme B (*GzmB*), Granzyme K (*GzmK*), and the transcription factor T-bet (*Tbx21*) (Fig. 3D). The expression of these genes was higher in OVA-specific CD8 T cells induced by OVA mRNA-LNP than in those by OVA protein + poly I/C. Furthermore, Perforin (*Prf1*) expression was also significantly induced in OVA-specific CD8 T cells induced by OVA-mRNA-LNP. Given that T-bet is a key transcription factor for IFNγ production, we examined the potential of the OVA-specific CD8 T cells to produce IFNγ in response to stimulation (Fig. 3E). In OVA mRNA-LNP-immunized mice, the proportion of IFNγ^+^ cells was significantly increased compared to the OVA + Poly I/C group. The frequency of IFNγ^+^ TNFα^+^ cells was comparable between groups, but in line with the increased OVA-specific CD8 T cell number, these cytokine-producing cells in the OVA mRNA-LNP group outnumbered those in the OVA protein + poly I/C group by approximately 5–6-fold. These data indicate that antigen-specific CD8 T cells induced by mRNA-LNP vaccination are functionally competent and exhibit enhanced effector potential.

OVA mRNA-LNP immunization resulted in significantly greater expression of programmed cell death protein 1 (PD-1) and lymphocyte activation gene 3 (LAG-3) (markers of TCR signals) on tetramer-positive CD8 T cells induced by OVA mRNA-LNP than those induced by OVA protein + poly I/C (Fig. 3, F and G).

These results indicate that mRNA-LNP elicits a significantly stronger and more sustained antigen-specific CD8 T cell response compared to conventional adjuvant formulations by providing a stronger TCR signal.

### TCR repertoire of CD8 T cells induced by mRNA-LNP

**Fig. 4.**
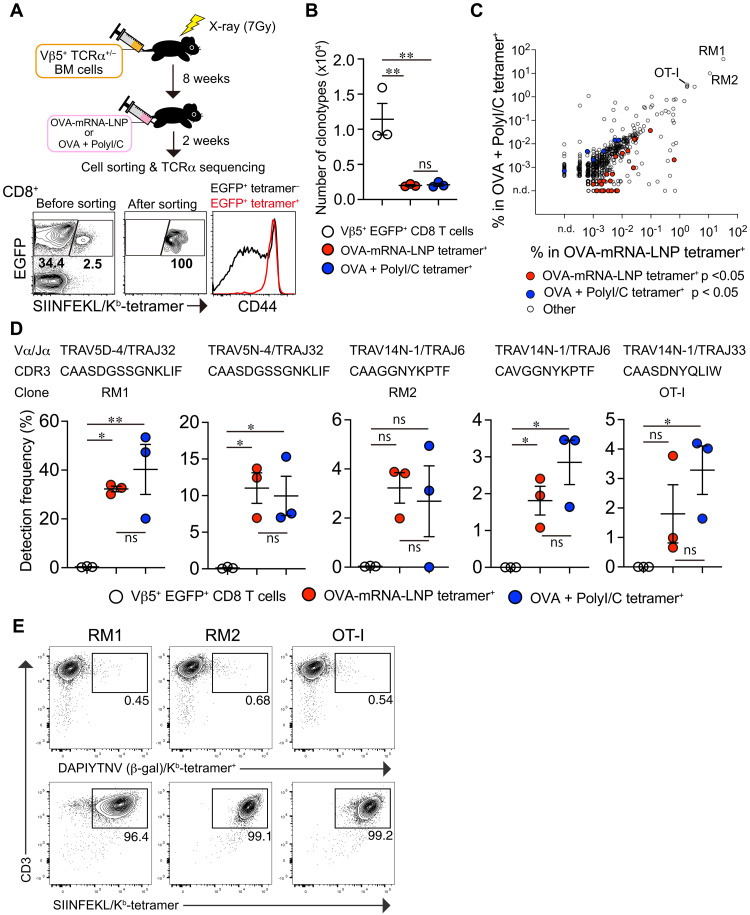
TCR repertoire analysis of OVA-specific CD8 T cells. (**A**) Schematic of TCRα repertoire analysis in OVA-specific CD8 T cells. TCRα^+/−^ bone marrow cells expressing TCR-Vβ5 chain (from OT-I TCR) and enhanced green fluorescent protein (EGFP) were intravenously transferred into irradiated WT mice. Eight weeks later, the recipient mice were immunized intramuscularly with OVA mRNA-LNP or OVA protein + poly I/C. Two weeks after immunization, EGFP^+^ Vβ5^+^ CD8 T cells (before immunization) or EGFP^+^ OVA-specific CD8 T cells from spleen of indicated mice were sorted and subjected to TCRα sequencing (*n* = 3 per group). Representative FACS plots before and after sorting, along with the CD44 expression profiles of the sorted cells, are shown. (**B**) The number of TCR clonotypes in EGFP^+^ Vβ5^+^ CD8 T cells (open circle), EGFP^+^ OVA-specific CD8 T cells (immunized with OVA-mRNA-LNP, red circle; immunized with OVA protein + poly I/C, blue circle) are shown. (**C**) Scatter plot of the detection frequency of TCRα chains in mice immunized with OVA mRNA-LNP or OVA protein + poly I/C. Clones detected at ≥0.0001% in ≥2 mice per group were shown. Clones significantly enriched in the OVA mRNA-LNP or OVA protein + poly I/C groups are shown in red and blue, respectively. (**D**)Vα/Jα segments and CDR3 amino acid sequences of the top five expanded TCR clones are shown. The graphs indicate the detection frequencies of these clones. (**E**) RM1, RM2, and OT-I TCRs were reconstituted in TG40 cells co-expressing CD8 and a set of CD3 subunits. These cells were examined for binding to SIINFEKL/K^b^-tetramer or DAPIYTNV (β-galactosidase: β-gal, an unrelated antigen) /K^b^-tetramer. Statistical significance was determined by one-way ANOVA with Dunnett’s multiple comparisons test [(B) and (D)], and two-tailed Student’s *t* test (C); *P < 0.05, **P < 0.01. ns, not significant.

To investigate whether mRNA-LNP affects the TCR repertoire of the induced CD8 T cells, we performed bulk TCR sequencing on the TCRα chains in retrogenic mice expressing a rearranged TCRβ chain (from the OT-I TCR). Retrogenic mice were immunized with OVA mRNA-LNP or OVA protein + poly I/C, and SIINFEKL/K^b^ tetramer-positive CD8 T cells (mostly CD44^hi^) were analyzed on day 14 (Fig. 4A). TCR sequencing analysis showed that the number of TCR clonotypes was markedly reduced in SIINFEKL/K^b^ tetramer-positive CD8 T cells compared to total CD8 T cells before immunization (Fig. 4B and fig. S5A). Scatter plot and principal component analysis revealed a marked similarity in the composition of OVA peptide-reactive TCRs between mice immunized with OVA mRNA-LNP and those receiving OVA protein + poly I/C (Fig. 4C and fig. S5B). In addition, significant differences in TCR clonotype frequency were limited to those with low frequencies. As shown in Fig. 4D, the frequencies of the top five expanded TCR clonotypes were not statistically different between the OVA mRNA-LNP and OVA + poly I/C groups. Of the five TCR clonotypes, three TCR clonotypes including OT-I reacted to SIINFEKL/K^b^ (Fig. 4E).

Taken together, the results showed similar TCR repertoires induced by OVA mRNA-LNP and OVA + poly I/C, suggesting that the robust expansion of CD8 T cells by mRNA-LNP is primarily due to differences that are quantitative (difference in magnitude) rather than qualitative (difference in types of T cells) compared to antigen protein and conventional adjuvants.

### mRNA-LNP induces potent and long-lasting antigen presentation in cDC2 cells

The activation of antigen-specific T cells depends on antigen-presenting cells that provide TCR signals. LNP itself causes inflammation and activates antigen-presenting cells (*9*, *20*). Indeed, the number of antigen-presenting cells such as mDCs and macrophages increased markedly in the dLN one day after mRNA intramuscular administration, regardless of whether mRNA was encapsulated in LNPs or not (fig. S6).

**Fig. 5.**
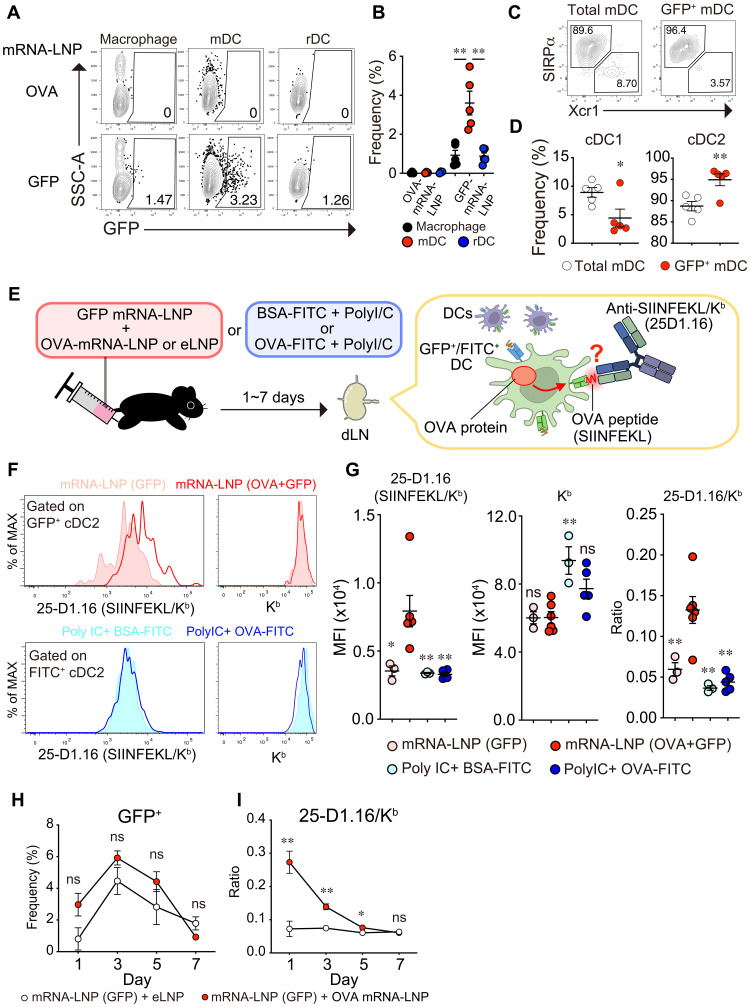
mRNA-LNP induces strong and durable antigen presentation. (**A** and **B**) GFP expression in macrophages, rDCs, and mDCs in the popliteal LNs of mice immunized with OVA-mRNA-LNP or GFP-mRNA-LNP on day 1 (A). The frequency of GFP^+^ cells among the indicated cell population (B). (*n* = 3–5 per group) (**C** and **D**) Expression of SIRPα and Xcr1 in total mDCs and GFP^+^ mDCs (C). Frequency of cDC1 cells (SIRPα^−^ and Xcr1^+^) and cDC2 cells (SIRPα^+^ and Xcr1^−^) within the indicated cell populations (D). (*n* = 5 per group) (**E**) C57BL/6 mice were intramuscularly administered GFP mRNA-LNP (1 μg) + OVA mRNA-LNP (1 μg) or GFP mRNA-LNP (1 μg) + LNP (eLNP, equivalent lipid amount). For comparison, mice were intramuscularly administered FITC-conjugated bovine serum albumin (BSA, 100 μg) + poly I/C (10 μg) or FITC-conjugated OVA (100 μg) + poly I/C (10 μg). OVA-derived SIINFEKL peptide–K^b^ complex on mDCs can be detected with the anti-SIINFEKL/K^b^ antibody (25D1.16). (**F**) The expression of SIINFEKL/K^b^ complexes and K^b^ in GFP^+^ or FITC^+^ cDC2 cells. Mice were treated as shown in (E) and analyzed one day after administration. (**G**) MFI of 25D1.16, K^b^ in GFP^+^ or FITC^+^ cDC2, and the ratio of 25D1.16/K^b^. mRNA-LNP (GFP), *n* = 3; mRNA-LNP (GFP + OVA), *n* = 4; BSA-FITC + poly I/C, *n* = 3; OVA-FITC + poly I/C, *n* = 5. (**H** and **I**) Time course of the frequency of GFP^+^ (H) and 25D1.16/K^b^ ratio (I) in cDC2 from mice administered GFP mRNA-LNP and OVA mRNA-LNP (*n* = 3) or eLNP (*n* = 2–3), as in (E). Statistical significance was determined by one-way ANOVA with Dunnett’s multiple comparisons test [(B) and (G)] or two-tailed Student’s *t* test for (D), (H), and (I); *P < 0.05, **P < 0.01. ns, not significant.

To identify antigen-presenting cells transduced by LNP, we used GFP-encoding mRNA encapsulated in LNP (GFP mRNA-LNP). One day after mice were intramuscularly injected with GFP mRNA-LNP, the expression of GFP was detected prominently in MHC class II^hi^ mDCs and weakly in macrophages and resident DCs (rDCs) in dLNs (Fig. 5, A and B). Among the mDC subsets, cDC2 cells (XCR1^−^ Sirpα^+^) were the main producers of GFP, indicating that intramuscularly administered LNPs are mainly taken up by migratory cDC2 cells (Fig. 5, C and D).

To explore the antigen-presenting ability of LNP-incorporated cDC2 cells, we co-injected GFP mRNA-LNP and OVA mRNA-LNP into mice, and measured the expression of MHC class I presenting OVA-derived peptide SIINFEKL/K^b^ in GFP-expressing cDC2 cells using the ‘TCR-like’ antibody 25D1.16, which allows detection of the peptide/MHC class I (pMHC-I) complex in vitro (*21*, *22*) (Fig. 5E). For comparison, fluorescein-conjugated OVA protein and poly I/C were injected. Upon co-injection of GFP mRNA-LNP + OVA mRNA-LNP, GFP-positive cDC2 cells displayed significantly increased SIINFEKL/K^b^ expression levels compared to those in mice injected with GFP-mRNA-LNP + eLNP (Fig. 5F). Such strong in vivo presentation of SIINFEKL/K^b^ was undetectable upon immunization with OVA protein. The ratio of expression level of SIINFEKL/K^b^ to total K^b^ was markedly higher in cDC2 cells from mRNA-LNP-immunized mice compared to control mice, but this ratio remained at control levels in mice immunized with OVA protein (Fig. 5G). The OVA protein used in this experiment was able to induce OVA-specific CD8 T cells (fig. S7), but the resulting antigen presentation was below the detection limit for the visualization of pMHC-I complexes in vivo. By contrast, OVA mRNA-LNP enabled the detection of pMHC-I complexes in vivo, indicating the extremely potent presentation of mRNA-encoded antigen peptide.

The time course of protein expression and antigen presentation after mRNA-LNP administration was also evaluated. GFP expression peaked on day 3, after which it decreased and became undetectable on day 7 (Fig. 5H). The presentation of SIINFEKL/K^b^ took a different course, peaking on day 1 and then gradually decreasing, remaining significantly higher than the control until day 5, and disappearing on day 7 (Fig. 5I). These results indicate that the MHC class I presentation of antigen peptide encoded by mRNA-LNP occurs prior to the accumulation of antigen protein and is sustained for up to 5 days in vivo.

### Antigen presentation by mRNA-LNP does not require cross-presentation

**Fig. 6.**
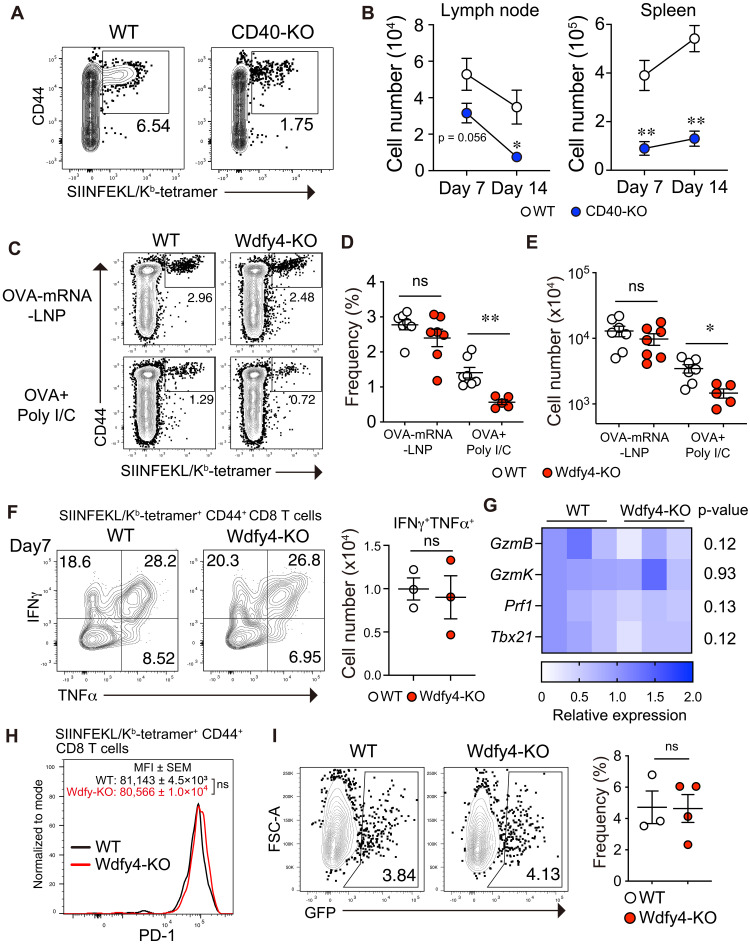
Antigen-specific CD8 T cell expansion induced by mRNA-LNP does not require cross-presentation. (**A**) Flow cytometry analysis of OVA-specific CD8 T cells (CD44^+^ SIINFEKL/K^b^-tetramer^+^) in the popliteal LNs of WT and CD40-deficient mice on day 7 after immunization. (**B**) Number of OVA-specific CD8 T cells in popliteal LNs and spleen of WT (*n* = 6) and CD40-deficient (*n* = 6–7) mice. (**C** to **E**) Flow cytometry analysis of OVA-specific CD8 T cells in the popliteal LNs of WT and Wdfy4-deficient mice on day 7 after immunization (C). Frequency (D) and cell number (E) of OVA-specific CD8 T cells. WT, *n* = 6–7; Wdfy4-deficient mice, *n* = 5–7. (**F**) Expression of IFNγ and TNFα in splenic OVA-specific CD8 T cells was analyzed by flow cytometry on day 7 after immunization. Number of IFNγ/TNFα double-positive OVA-specific CD8 T cells in the indicated mice (*n* = 3 per group). (**G**) Heatmap of *GzmB*, *GzmK*, *Prf1*, and *Tbx21* mRNA expression in sorted CD44^hi^ tetramer^+^ OVA-specific CD8 T cells from spleens of the indicated mice at day 7 after immunization, measured by qRT-PCR (*n* = 3 per group). (**H**) PD-1 expression in OVA-specific CD8 T cells from the LNs on day 7 after OVA mRNA-LNP immunization. The mean ± SEM of PD-1 MFI is shown (*n* = 3 per group). (**I**) GFP expression in mDCs in the popliteal LNs of WT and Wdfy4-deficient mice immunized with GFP-mRNA-LNP on day 1. The graphs indicate the frequency of OVA-GFP+ cells among mDCs in WT (*n* = 3) and Wdfy4-deficient mice (*n* = 4). Statistical significance was determined by two-tailed Student’s *t* test for (B) and (D) to (I); *P < 0.05, **P < 0.01. ns, not significant.

The survival of mDCs during antigen presentation requires CD40 signaling (*23*). Our results showed that, in CD40-deficient mice, the number of OVA-specific CD8 T cells in dLNs was significantly lower than that in wild-type mice on day 7 after OVA mRNA-LNP administration, and further decreased on day 14 (Fig. 6, A and B). Consequently, the number of OVA-specific CD8 T cells in the spleen was also markedly lower in CD40-deficient mice. These data suggest that mRNA-LNP requires sustained antigen presentation by mDCs to achieve the high induction of CD8 T cells.

A previous study utilizing mice administered the BNT162b2 mRNA vaccine suggested that the cross-presentation pathway is important for inducing CD8 T cell responses (*8*). However, our present results indicate that mRNA-LNPs are primarily taken up by cDC2 cells, rather than the cDC1 cells typically associated with efficient cross-presentation of soluble antigens, and induce antigen presentation prior to the production of antigen protein. To determine whether the cross-presentation pathway is involved in CD8 T cell induction by mRNA-LNPs, we utilized mice deficient in WD repeat and FYVE domain-containing protein 4 (Wdfy4), a protein essential for cross-presentation (*24*). Consistent with a recent report (*25*), Wdfy4-deficient mice exhibited significantly reduced CD8 T cell induction in response to immunization with OVA protein and poly I/C (Fig. 6, C to E). By contrast, when immunized with OVA mRNA-LNP, the induction of OVA-specific CD8 T cells was unaffected in Wdfy4-deficient mice. Cytokine production (Fig. 6F), cytotoxic molecule expression (Fig. 6G), and PD-1 levels in OVA-specific CD8 T cells (Fig. 6H) were comparable between WT and Wdfy4-deficient mice. The deficiency of Wdfy4 did not affect the ability to uptake LNPs by DC subsets, as GFP expression in cDC1 and cDC2 cells was similar in Wdfy4-deficient mice and control mice immunized with GFP mRNA-LNP (Fig. 6I). These data indicate that cross-presentation is not required for the robust induction of CD8 T cells by mRNA-LNP, suggesting the role of the unconventional antigen presentation pathway in the immune responses induced by mRNA vaccines.

## DISCUSSION

This study elucidated the mechanism of action of the mRNA-LNP formulation by comparing the immune responses induced by mRNA-LNP with those induced by conventional adjuvants and antigenic proteins. Our results showed that mRNA-LNP induced the production of IgG2b and IgG2c rather than IgG1 antibodies due to the skewed differentiation of Th cells towards Th1. mRNA-LNP also induced potent and durable antigen presentation via MHC class I, mainly by cDC2 cells, which in turn induced the robust expansion of antigen-specific CD8 T cells. These characteristics of immune responses are quantitatively and qualitatively different from those induced by conventional adjuvants such as Toll-like receptor ligands. This study focused on immune responses against OVA, and thus further investigation is needed to evaluate the efficacy of mRNA-LNP formulations using clinically relevant antigens such as those from SARS-CoV-2 and influenza virus. It will also be important to compare the humoral and cellular immune responses elicited by mRNA-LNPs with different RNA modifications.

The high production of antigen-specific IgG2b and IgG2c antibodies in mice following mRNA-LNP administration may be due to a mechanism similar to the increased IgG4 production observed in humans receiving repeated mRNA vaccinations. While it is widely recognized that mRNA vaccines used against COVID-19 can induce high antibody production, several studies have reported the marked production of antigen-specific IgG4 in response to repeated vaccination (*14*–*16*). IgG4 is an immunosuppressive subclass of antibody with low affinity for Fc receptors and complement component C1q, and is likely to reduce protection against SARS-CoV-2 infection (*26*, *27*). The Cγ genes that encode the constant region of IgG are located on the human chromosome in the order of Cγ3 (IgG3), Cγ1 (IgG1), Cγ2 (IgG2), and Cγ4 (IgG4); or on the mouse chromosome in the order of Cγ3 (IgG3), Cγ1 (IgG1), Cγ2a (IgG2a), Cγ2b (IgG2b), and Cγ2c (IgG2c). Mouse IgG2c is a distal IgG subclass that corresponds to human IgG4, with both located most distally from the VDJ region within the immunoglobulin heavy-chain locus. It is possible that the production of large amounts of antigens by mRNA-LNP induces longer-lasting immune responses than with conventional component vaccines or viral infections, promoting class switch recombination and subsequent production of IgG4 in human (IgG2c in mouse). Human IgG4 and mouse IgG2c are functionally distinct, but their production may be regulated by a shared mechanism in that both are generated by enhanced class switching. Our data indicate that IgG2c production induced by mRNA-LNP in mice requires enhanced Th1 differentiation and IFNγ signaling. Although the precise mechanism by which mRNA-LNP induces Th1 differentiation remains unclear, it is possible that innate sensing of double-stranded RNA through MDA5 or RIG-I enhances Th1 responses, even when using modified mRNA (*28*). Further studies are needed to investigate whether the differentiation of Th cells can be reproduced using mRNA-LNP with human DCs and T cells.

Presentation of antigens encoded by mRNA-LNP is mediated mainly by cDC2 cells, which differs from conventional component vaccines and viral infections that are mainly driven by cDC1 cells. It has been previously reported that LNP is readily incorporated into cDC2 cells (*13*), but the mechanism remains unclear. A recent study has suggested that intramuscularly administered mRNA-LNPs reach draining lymph nodes where they are incorporated into cDC2 cells (*29*). On the other hand, it is important to evaluate the contribution of cDC2 cells in the local sites to the efficacy of mRNA-LNPs, for example using genetically modified mice such as DC-specific CCR7-deficient mice. Our results showed that cDC2 cells incorporating mRNA-LNP exhibited strong antigen presentation by MHC class I for long periods of time, which is normally undetectable in vivo. This is the reason why mRNA-LNP induces strong and durable CD8 T cell responses. In CD40-deficient mice, the CD8 T cell response induced by mRNA-LNP is weakened as the survival of DCs is impaired (*23*). In addition, the CD8 T cell response induced by mRNA-LNP does not require cross-presentation.

Cross-presentation is a key pathway by which cDC1 cells present soluble antigens or antigens derived from bacteria, viruses, and tumor cells via MHC class I to prime antigen-specific CD8 T cells. Antigens endocytosed by cDC1 cells are exported from endosomes to the cytosol, where they are degraded by proteasomes, and the resulting peptides are presented on the cell surface via MHC class I. Previously reported key factors for cross-presentation include perforin 2 and Wdfy4, although the mechanism remains largely unknown (*24*, *30*). Our results clearly show that Wdfy4-dependent cross-presentation is crucial for CD8 T cell responses elicited by soluble antigens, but not for those induced by mRNA-LNP. The OVA antigen used in this study is a secreted soluble protein. Thus, while cDC1 cells may endocytose and cross-present extracellular OVA proteins, this mechanism contributes minimally to mRNA-LNP-induced CD8 T cell responses. Presentation of the peptide encoded by mRNA-LNP via MHC class I reaches its maximum level before the protein expression peaked. This is consistent with the results that cross-presentation of extracellular proteins is not required for the mRNA-LNP-induced CD8 T cell expansion. Rather, it is suggested that the proteins synthesized from mRNA within the LNP are immediately degraded by proteasomes and presented by MHC class I.

The rapid degradation of a fraction of the proteins during ribosomal translation and their contribution to antigen presentation by MHC class I are known as the defective ribosomal products (DRiPs) pathway (*31*, *32*). This mechanism plays an important role in response to viral infection, enabling the presentation of virus-derived peptides to cytotoxic CD8 T cells before the synthesis of viral proteins and the formation of viral particles (*33*, *34*). However, the molecular mechanisms that control the DRiPs pathway remain unclear. Further exploration of the molecules involved in antigen presentation by mRNA-LNP will greatly advance our understanding of the molecular mechanisms of the DRiPs pathway as well as the molecular basis that links protein metabolism and antigen presentation.

Taken together, this study showed that the mRNA-LNP formulation, unlike conventional adjuvant-based immunization, induces Th1-biased CD4 T cell differentiation, followed by the production of class-switched IgG, and causes the long-term expansion of CD8 T cells. The potent and durable presentation of antigens encoded by mRNA-LNP, combined with the adjuvanticity of the LNP components to induce alarmin responses, may lead to the induction of unconventional T cell responses. A total of 13 billion mRNA-LNP vaccine doses targeting SARS-CoV-2 have been administered worldwide due to their strong immunogenicity. On the other hand, some individuals experience adverse events, including a constellation of chronic symptoms sometimes referred to post-vaccination syndrome. In rare instances, these events can be severe or even fatal. Dissecting the pathogenesis of post-vaccination syndrome using appropriate mouse models is an extremely important and timely goal for future research (*20*). Even four years after mass vaccination, the mechanism of action and long-term effects of mRNA-LNP have not yet been clarified. Understanding the molecular and immunological actions of mRNA-LNP formulations through rigorous studies is crucial for advancing medical technology and establishing sound public health policies.

## MATERIALS AND METHODS

### Mice

C57BL/6 N mice were purchased from Japan SLC Inc. (Shizuoka, Japan). TCRα^−/−^ mice (*35*), CD40^−/−^ mice (*36*), IFNγR1^−/−^ mice (*37*), CD4Cre mice (*38*), and Bcl6^flox^ mice (*39*) were previously described. Wdfy4^−/−^ mice were generated by the CRISPR/Cas9-mediated genome editing, with the following target sequence: ATCTGCTTCGCCAACGATGG. All mice were bred and maintained under specific pathogen-free conditions in our animal facility and were euthanized by overdose of inhalation anesthetics. Animal experiments were approved by the President of the Tokyo University of Science (S24002) and University of Tokyo (I-P21–110) and the JIHS following review by the respective Institutional Animal Care and Use Committee (2023-A045). All procedures were conducted in accordance with institutional procedures, national guidelines, and the relevant national laws on the protection of animals.

### mRNA-LNP

Five-methoxyuridine-modified mRNA (5-moU) encoding soluble OVA (#MRNA-41) or GFP (#MRNA-11) were purchased from OZ Biosciences (Marseille, France) and encapsulated in LNPs of the composition identical to the Pfizer/BioNTech mRNA vaccine BNT162b2 by Katayama Chemical Industries Co., Ltd. (Osaka, Japan). Empty LNPs without encapsulating mRNA were also produced by Katayama Chemical Industries Co., Ltd.

### Immunization

Mice aged 8–12 weeks were injected intramuscularly with 30 μl mRNA-LNPs (containing 1 μg mRNA) under anesthesia. For comparison, 100 μg OVA protein (Sigma-Aldrich, St. Louis, MO, USA) and 10 μg poly I/C (InvivoGen, San Diego, CA, USA), 10 μg LPS (Sigma-Aldrich), or 15 μl AddaVax (InvivoGen) were injected. If necessary, administration was repeated on days 14 and 28.

### Enzyme-linked immunosorbent assay

Flat-bottom 96-well plates (Thermo Fisher Scientific, Waltham, MA, USA) were coated with 10 μg/ml OVA (Sigma-Aldrich) and incubated overnight at 4°C. After washing the plates three times with phosphate-buffered saline (PBS) containing 0.05% Tween-20 (PBST), wells were blocked with PBS containing 1% bovine serum albumin for 1 hour at room temperature. Subsequently, 50 μl diluted serum samples (typically 1:10^3^ to 1:10^5^) were added to each well and incubated at room temperature for 1 hour. Plates were washed three times with PBST, and 50 μl HRP-conjugated anti-mouse IgG, IgG1, IgG2b, or IgG2c antibodies (diluted 1:5000; SouthernBiotech, Birmingham, AL, USA) were added to the wells. After three washes with PBST, 50 μl TMB Substrate Solution (Thermo Fisher Scientific) was added and allowed to react for 15 min. The reaction was stopped by the addition of 2 N sulfuric acid. Absorbance was measured at 450 nm with a reference wavelength of 570 nm using the GloMax Discover Microplate Reader (Promega, Madison, WI, USA). To estimate antigen-specific IgG titers, serially diluted serum samples were tested, and the highest dilution that yielded an absorbance value between 0.1 and 1.0 was defined as the IgG titer.

### Flow cytometry

Flow cytometry analysis and cell sorting were performed with the FACSCanto II and FACSAria III systems (BD Biosciences, Franklin, NJ, USA). LN cells were prepared by digesting isolated LNs with 0.2% Collagenase D (Roche, Basel, Switzerland) and 0.01% DNase I (Roche). Prior to cell staining, the Fc blocker (anti-mouse CD16/CD32; clone 2.4G2; TONBO Biosciences, San Diego, CA, USA) was used. Cells were stained with a mixture of the antibodies at a final concentration of 1 to 5 μg/ml. 7-Aminoactinomycin D was used to exclude dead cells. For pMHC tetramer staining, cells were incubated with PE-conjugated SIINFEKL/K^b^ (MBL) for 30–60 min at 4°C, and then stained with fluorescence-labeled antibodies. For cytokine production, cells were incubated in RPMI 1640 complete medium in the presence of phorbol myristate acetate (PMA, 2.5 ng/ml), ionomycin (1 μg/ml) and brefeldin A (1 μg/ml) at 37°C for 4 hours. After cell surface staining, cells were fixed using a Foxp3 Staining Buffer Set (eBioscience). For detection of OVA-reactive germinal center B cells, LN cells were incubated with recombinant OVA labeled with Alexa Fluor 647 (Invitrogen, Thermo Fisher Scientific) on ice for 30 min prior to surface staining.

### TCR repertoire analysis

Retrogenic mice were generated as previously described (*40*, *41*), using a retrovirus vector expressing TCRβ (Vβ5.2-Dβ2-Jβ2.6-Cβ2 from OT-I-TCR). GFP^+^ tetramer^+^ or GFP^+^ tetramer^−^ CD8 T cells were purified from retrogenic mice, and total RNA was extracted with ISOGEN (Nippon Gene, Tokyo, Japan). Next-generation sequencing with an unbiased TCR repertoire analysis technology was performed by Repertoire Genesis Inc. (Osaka, Japan). The sequencing data have been deposited in the DDBJ Sequence Read Archive under BioProject accession PRJDB40453. The source code for principal component analysis of TCR frequency is available in Dryad (https://doi.org/10.5061/dryad.h44j0zq25) and GitHub (https://github.com/nittatakeshi).

### Quantitative mRNA analysis

Total RNA was extracted from sorted cells using the RNeasy Micro Kit (QIAGEN) and reverse transcribed with Superscript III (Invitrogen, Thermo Fisher Scientific). Quantitative PCR was performed with SYBR Green Realtime PCR Master Mix (TOYOBO) and the StepOne Real-Time PCR System (Life Technologies, Thermo Fisher Scientific). The results were normalized to the Gapdh or β-actin expression levels.

### Statistical analyses

Statistical significance was calculated with the unpaired two-tailed Student’s *t* test using GraphPad Prism 10 (GraphPad Software, La Jolla, CA, USA). No randomization or exclusion of samples was used. For animal experiments, sample size was chosen based on previous experience and preliminary experiments.

## References

[1] T. L. Murphy, G. E. Grajales-Reyes, X. Wu, R. Tussiwand, C. G. Briseño, A. Iwata, N. M. Kretzer, V. Durai, K. M. Murphy, Transcriptional control of dendritic cell development. Annu. Rev. Immunol. 34, 93–119 (2016). 26735697 10.1146/annurev-immunol-032713-120204 PMC5135011

[2] V. Durai, K. M. Murphy, Functions of murine dendritic cells. Immunity 45, 719–736 (2016). 27760337 10.1016/j.immuni.2016.10.010 PMC5145312

[3] O. P. Joffre, E. Segura, A. Savina, S. Amigorena, Cross-presentation by dendritic cells. Nat. Rev. Immunol. 12, 557–569 (2012). 22790179 10.1038/nri3254

[4] J. T. Granados-Riveron, G. Aquino-Jarquin, Engineering of the current nucleoside-modified mRNA-LNP vaccines against SARS-CoV-2. Biomed. Pharmacother. 142, 111953 (2021). 34343897 10.1016/j.biopha.2021.111953 PMC8299225

[5] M. J. Hogan, N. Pardi, mRNA vaccines in the COVID-19 pandemic and beyond. Annu. Rev. Med. 73, 17–39 (2022). 34669432 10.1146/annurev-med-042420-112725

[6] N. Chaudhary, D. Weissman, K. A. Whitehead, mRNA vaccines for infectious diseases: Principles, delivery and clinical translation. Nat. Rev. Drug Discov. 20, 817–838 (2021). 34433919 10.1038/s41573-021-00283-5 PMC8386155

[7] U. Sahin, A. Muik, I. Vogler, E. Derhovanessian, L. M. Kranz, M. Vormehr, J. Quandt, N. Bidmon, A. Ulges, A. Baum, K. E. Pascal, D. Maurus, S. Brachtendorf, V. Lörks, J. Sikorski, P. Koch, R. Hilker, D. Becker, A. K. Eller, J. Grützner, M. Tonigold, C. Boesler, C. Rosenbaum, L. Heesen, M. C. Kühnle, A. Poran, J. Z. Dong, U. Luxemburger, A. Kemmer-Brück, D. Langer, M. Bexon, S. Bolte, T. Palanche, A. Schultz, S. Baumann, A. J. Mahiny, G. Boros, J. Reinholz, G. T. Szabó, K. Karikó, P.-Y. Shi, C. Fontes-Garfias, J. L. Perez, M. Cutler, D. Cooper, C. A. Kyratsous, P. R. Dormitzer, K. U. Jansen, Ö. Türeci, BNT162b2 vaccine induces neutralizing antibodies and poly-specific T cells in humans. Nature 595, 572–577 (2021). 34044428 10.1038/s41586-021-03653-6

[8] C. Li, A. Lee, L. Grigoryan, P. S. Arunachalam, M. K. D. Scott, M. Trisal, F. Wimmers, M. Sanyal, P. A. Weidenbacher, Y. Feng, J. Z. Adamska, E. Valore, Y. Wang, R. Verma, N. Reis, D. Dunham, R. O’Hara, H. Park, W. Luo, A. D. Gitlin, P. Kim, P. Khatri, K. C. Nadeau, B. Pulendran, Mechanisms of innate and adaptive immunity to the Pfizer-BioNTech BNT162b2 vaccine. Nat. Immunol. 23, 543–555 (2022). 35288714 10.1038/s41590-022-01163-9 PMC8989677

[9] M. G. Alameh, I. Tombácz, E. Bettini, K. Lederer, C. Sittplangkoon, J. R. Wilmore, B. T. Gaudette, O. Y. Soliman, M. Pine, P. Hicks, T. B. Manzoni, J. J. Knox, J. L. Johnson, D. Laczkó, H. Muramatsu, B. Davis, W. Meng, A. M. Rosenfeld, S. Strohmeier, P. J. C. Lin, B. L. Mui, Y. K. Tam, K. Karikó, A. Jacquet, F. Krammer, P. Bates, M. P. Cancro, D. Weissman, E. T. Luning Prak, D. Allman, M. Locci, N. Pardi, Lipid nanoparticles enhance the efficacy of mRNA and protein subunit vaccines by inducing robust T follicular helper cell and humoral responses. Immunity 55, 1136–1138 (2022). 35704995 10.1016/j.immuni.2022.05.007 PMC9195404

[10] K. Kobiyama, K. J. Ishii, Making innate sense of mRNA vaccine adjuvanticity. Nat. Immunol. 23, 474–476 (2022). 35354958 10.1038/s41590-022-01168-4

[11] B. Z. Igyártó, Z. Qin, The mRNA-LNP vaccines - the good, the bad and the ugly? Front. Immunol. 15, 1336906 (2024). 38390323 10.3389/fimmu.2024.1336906 PMC10883065

[12] U. Sahin, A. Muik, E. Derhovanessian, I. Vogler, L. M. Kranz, M. Vormehr, A. Baum, K. Pascal, J. Quandt, D. Maurus, S. Brachtendorf, V. Lörks, J. Sikorski, R. Hilker, D. Becker, A. K. Eller, J. Grützner, C. Boesler, C. Rosenbaum, M. C. Kühnle, U. Luxemburger, A. Kemmer-Brück, D. Langer, M. Bexon, S. Bolte, K. Karikó, T. Palanche, B. Fischer, A. Schultz, P.-Y. Shi, C. Fontes-Garfias, J. L. Perez, K. A. Swanson, J. Loschko, I. L. Scully, M. Cutler, W. Kalina, C. A. Kyratsous, D. Cooper, P. R. Dormitzer, K. U. Jansen, Ö. Türeci, COVID-19 vaccine BNT162b1 elicits human antibody and T_H_1 T cell responses. Nature 586, 594–599 (2020). 32998157 10.1038/s41586-020-2814-7

[13] R. Oyama, H. Ishigame, H. Tanaka, N. Tateshita, M. Itazawa, R. Imai, N. Nishiumi, J. I. Kishikawa, T. Kato, J. Anindita, Y. Nishikawa, M. Maeki, M. Tokeshi, K. Tange, Y. Nakai, Y. Sakurai, T. Okada, H. Akita, An ionizable lipid material with a vitamin E scaffold as an mRNA vaccine platform for efficient cytotoxic T cell responses. ACS Nano 17, 18758–18774 (2023). 37814788 10.1021/acsnano.3c02251 PMC10569098

[14] J. S. Buhre, T. Pongracz, I. Künsting, A. S. Lixenfeld, W. Wang, J. Nouta, S. Lehrian, F. Schmelter, H. B. Lunding, L. Dühring, C. Kern, J. Petry, E. L. Martin, B. Föh, M. Steinhaus, V. von Kopylow, C. Sina, T. Graf, J. Rahmöller, M. Wuhrer, M. Ehlers, mRNA vaccines against SARS-CoV-2 induce comparably low long-term IgG Fc galactosylation and sialylation levels but increasing long-term IgG4 responses compared to an adenovirus-based vaccine. Front. Immunol. 13, 1020844 (2023). 36713457 10.3389/fimmu.2022.1020844 PMC9877300

[15] P. Irrgang, J. Gerling, K. Kocher, D. Lapuente, P. Steininger, K. Habenicht, M. Wytopil, S. Beileke, S. Schäfer, J. Zhong, G. Ssebyatika, T. Krey, V. Falcone, C. Schülein, A. S. Peter, K. Nganou-Makamdop, H. Hengel, J. Held, C. Bogdan, K. Überla, K. Schober, T. H. Winkler, M. Tenbusch, Class switch toward noninflammatory, spike-specific IgG4 antibodies after repeated SARS-CoV-2 mRNA vaccination. Sci. Immunol. 8, eade2798 (2023). 36548397 10.1126/sciimmunol.ade2798 PMC9847566

[16] P. Kiszel, P. Sík, J. Miklós, E. Kajdácsi, G. Sinkovits, L. Cervenak, Z. Prohászka, Class switch towards spike protein-specific IgG4 antibodies after SARS-CoV-2 mRNA vaccination depends on prior infection history. Sci. Rep. 13, 13166 (2023). 37574522 10.1038/s41598-023-40103-x PMC10423719

[17] K. Miyauchi, A. Sugimoto-Ishige, Y. Harada, Y. Adachi, Y. Usami, T. Kaji, K. Inoue, H. Hasegawa, T. Watanabe, A. Hijikata, S. Fukuyama, T. Maemura, M. Okada-Hatakeyama, O. Ohara, Y. Kawaoka, Y. Takahashi, T. Takemori, M. Kubo, Protective neutralizing influenza antibody response in the absence of T follicular helper cells. Nat. Immunol. 17, 1447–1458 (2016). 27798619 10.1038/ni.3563

[18] H. Liang, J. Tang, Z. Liu, Y. Liu, Y. Huang, Y. Xu, P. Hao, Z. Yin, J. Zhong, L. Ye, X. Jin, H. Wang, ZIKV infection induces robust Th1-like Tfh cell and long-term protective antibody responses in immunocompetent mice. Nat. Commun. 10, 3859 (2019). 31455769 10.1038/s41467-019-11754-0 PMC6712032

[19] V. Oberhardt, H. Luxenburger, J. Kemming, I. Schulien, K. Ciminski, S. Giese, B. Csernalabics, J. Lang-Meli, I. Janowska, J. Staniek, K. Wild, K. Basho, M. S. Marinescu, J. Fuchs, F. Topfstedt, A. Janda, O. Sogukpinar, H. Hilger, K. Stete, F. Emmerich, B. Bengsch, C. F. Waller, S. Rieg, Sagar, T. Boettler, K. Zoldan, G. Kochs, M. Schwemmle, M. Rizzi, R. Thimme, C. Neumann-Haefelin, M. Hofmann, Rapid and stable mobilization of CD8^+^ T cells by SARS-CoV-2 mRNA vaccine. Nature 597, 268–273 (2021). 34320609 10.1038/s41586-021-03841-4 PMC8426185

[20] S. Tahtinen, A. J. Tong, P. Himmels, J. Oh, A. Paler-Martinez, L. Kim, S. Wichner, Y. Oei, M. J. McCarron, E. C. Freund, Z. A. Amir, C. C. de la Cruz, B. Haley, C. Blanchette, J. M. Schartner, W. Ye, M. Yadav, U. Sahin, L. Delamarre, I. Mellman, IL-1 and IL-1ra are key regulators of the inflammatory response to RNA vaccines. Nat. Immunol. 23, 532–542 (2022). 35332327 10.1038/s41590-022-01160-y

[21] A. Porgador, J. W. Yewdell, Y. Deng, J. R. Bennink, R. N. Germain, Localization, quantitation, and in situ detection of specific peptide-MHC class I complexes using a monoclonal antibody. Immunity 6, 715–726 (1997). 9208844 10.1016/s1074-7613(00)80447-1

[22] T. Mareeva, E. Martinez-Hackert, Y. Sykulev, How a T cell receptor-like antibody recognizes major histocompatibility complex-bound peptide. J. Biol. Chem. 283, 29053–29059 (2008). 18703505 10.1074/jbc.M804996200 PMC2570882

[23] R. Wu, R. A. Ohara, S. Jo, T.-T. Liu, S. T. Ferris, F. Ou, S. Kim, D. J. Theisen, D. A. Anderson III, B. W. Wong, T. Gershon, R. D. Schreiber, T. L. Murphy, K. M. Murphy, Mechanisms of CD40-dependent cDC1 licensing beyond costimulation. Nat. Immunol. 23, 1536–1550 (2022). 36271147 10.1038/s41590-022-01324-w PMC9896965

[24] D. J. Theisen, J. T. Davidson IV, C. G. Briseño, M. Gargaro, E. J. Lauron, Q. Wang, P. Desai, V. Durai, P. Bagadia, J. R. Brickner, W. L. Beatty, H. W. Virgin, W. E. Gillanders, N. Mosammaparast, M. S. Diamond, L. D. Sibley, W. Yokoyama, R. D. Schreiber, T. L. Murphy, K. M. Murphy, WDFY4 is required for cross-presentation in response to viral and tumor antigens. Science 362, 694–699 (2018). 30409884 10.1126/science.aat5030 PMC6655551

[25] S. Jo, R. A. Ohara, D. J. Theisen, S. Kim, T. Liu, C. B. Bullock, M. He, F. Ou, J. Chen, S. J. Piersma, J. L. Postoak, W. M. Yokoyama, M. S. Diamond, T. L. Murphy, K. M. Murphy, Shared pathway of WDFY4-dependent cross-presentation of immune complexes by cDC1 and cDC2. J. Exp. Med. 222, e20240955 (2025). 39918736 10.1084/jem.20240955 PMC11804880

[26] V. N. Uversky, E. M. Redwan, W. Makis, A. Rubio-Casillas, IgG4 antibodies induced by repeated vaccination may generate immune tolerance to the SARS-CoV-2 spike protein. Vaccines 11, 991 (2023). 37243095 10.3390/vaccines11050991 PMC10222767

[27] C. Martín Pérez, S. Ruiz-Rius, A. Ramírez-Morros, M. Vidal, D. H. Opi, P. Santamaria, J. Blanco, J. Vidal-Alaball, J. G. Beeson, L. M. Molinos-Albert, R. Aguilar, A. Ruiz-Comellas, G. Moncunill, C. Dobaño, Post-vaccination IgG4 and IgG2 class switch associates with increased risk of SARS-CoV-2 infections. J. Infect. 90, 106473 (2025). 40113142 10.1016/j.jinf.2025.106473

[28] R. Verbeke, M. J. Hogan, K. Loré, N. Pardi, Innate immune mechanisms of mRNA vaccines. Immunity 55, 1993–2005 (2022). 36351374 10.1016/j.immuni.2022.10.014 PMC9641982

[29] D. Castaño, E. Bettini, B. Kumar, A. Chudnovskiy, A. Siv, G. Protti, S. Nakadakari-Higa, S. Ceglia, N. De Luna, J. E. Chiu, K. Lederer, S. H. Li, H. Ibrahim, H. Muramatsu, T. Mdluli, E. Abraham, S. E. Sahingur, I. Maillard, Y. K. Tam, S. Shin, S. E. Hensley, J. J. Miner, Z. Lipinszki, A. Reboldi, N. Pardi, R. Spreafico, G. D. Victora, M. Locci, Distinct components of mRNA vaccines cooperate to instruct efficient germinal center responses. Cell 188, 7461–7480.e23 (2025). 41406961 10.1016/j.cell.2025.11.023 PMC12878702

[30] P. Rodríguez-Silvestre, M. Laub, P. A. Krawczyk, A. K. Davies, J. P. Schessner, R. Parveen, B. J. Tuck, W. A. McEwan, G. H. H. Borner, P. Kozik, Perforin-2 is a pore-forming effector of endocytic escape in cross-presenting dendritic cells. Science 380, 1258–1265 (2023). 37347855 10.1126/science.adg8802 PMC7614779

[31] J. W. Yewdell, E. Reits, J. Neefjes, Making sense of mass destruction: Quantitating MHC class I antigen presentation. Nat. Rev. Immunol. 3, 952–961 (2003). 14647477 10.1038/nri1250

[32] K. L. Rock, D. J. Farfán-Arribas, J. D. Colbert, A. L. Goldberg, Re-examining class-I presentation and the DRiP hypothesis. Trends Immunol. 35, 144–152 (2014). 24566257 10.1016/j.it.2014.01.002 PMC3986829

[33] U. Schubert, L. C. Antón, J. Gibbs, C. C. Norbury, J. W. Yewdell, J. R. Bennink, Rapid degradation of a large fraction of newly synthesized proteins by proteasomes. Nature 404, 770–774 (2000). 10783891 10.1038/35008096

[34] E. A. Reits, J. C. Vos, M. Grommé, J. Neefjes, The major substrates for TAP in vivo are derived from newly synthesized proteins. Nature 404, 774–778 (2000). 10783892 10.1038/35008103

[35] P. Mombaerts, A. R. Clarke, M. A. Rudnicki, J. Iacomini, S. Itohara, J. J. Lafaille, L. Wang, Y. Ichikawa, R. Jaenisch, M. L. Hooper, S. Tonegawa, Mutations in T-cell antigen receptor genes α and β block thymocyte development at different stages. Nature 360, 225–231 (1992). 1359428 10.1038/360225a0

[36] T. Kawabe, T. Naka, K. Yoshida, T. Tanaka, H. Fujiwara, S. Suematsu, N. Yoshida, T. Kishimoto, H. Kikutani, The immune responses in CD40-deficient mice: Impaired immunoglobulin class switching and germinal center formation. Immunity 1, 167–178 (1994). 7534202 10.1016/1074-7613(94)90095-7

[37] S. Huang, W. Hendriks, A. Althage, S. Hemmi, H. Bluethmann, R. Kamijo, J. Vilček, R. M. Zinkernagel, M. Aguet, Immune response in mice that lack the Interferon-γ receptor. Science 259, 1742–1745 (1993). 8456301 10.1126/science.8456301

[38] P. P. Lee, D. R. Fitzpatrick, C. Beard, H. K. Jessup, S. Lehar, K. W. Makar, M. Pérez-Melgosa, M. T. Sweetser, M. S. Schlissel, S. Nguyen, S. R. Cherry, J. H. Tsai, S. M. Tucker, W. M. Weaver, A. Kelso, R. Jaenisch, C. B. Wilson, A critical role for Dnmt1 and DNA methylation in T cell development, function, and survival. Immunity 15, 763–774 (2001). 11728338 10.1016/s1074-7613(01)00227-8

[39] T. Kaji, A. Ishige, M. Hikida, J. Taka, A. Hijikata, M. Kubo, T. Nagashima, Y. Takahashi, T. Kurosaki, M. Okada, O. Ohara, K. Rajewsky, T. Takemori, Distinct cellular pathways select germline-encoded and somatically mutated antibodies into immunological memory. J. Exp. Med. 209, 2079–2097 (2012). 23027924 10.1084/jem.20120127 PMC3478929

[40] T. Nitta, Y. Kochi, R. Muro, Y. Tomofuji, T. Okamura, S. Murata, H. Suzuki, T. Sumida, K. Yamamoto, H. Takayanagi, Human thymoproteasome variations influence CD8 T cell selection. Sci. Immunol. 2, eaan5165 (2017). 28783658 10.1126/sciimmunol.aan5165

[41] R. Muro, H. Takayanagi, T. Nitta, Retroviral gene transduction into t cell progenitors for analysis of T cell development in the thymus. Methods Mol. Biol. 2111, 193–203 (2020). 31933209 10.1007/978-1-0716-0266-9_16

