# Supplementary material for: A distinct antigen presentation pathway drives potent T cell immunity in lipid nanoparticle–based mRNA vaccines
Source: Sci Adv. 2026 Jul 17;12(29):eaec7827. doi: 10.1126/sciadv.aec7827 (PMC13378541; doi:10.1126/sciadv.aec7827)
Supplement: Supplementary file 1 — Figs. S1 to S7 Legends for data S1 and S2 [file sciadv.aec7827_sm.pdf]

Science Advances
AAAS

Supplementary Materials for

A distinct antigen presentation pathway drives potent T cell immunity in lipid nanoparticle–based mRNA vaccines

Ryunosuke Muro *et al.*

Corresponding author: Takeshi Nitta, nittatakeshi@rs.tus.ac.jp; Hiroshi Takayanagi, takayana@m.u-tokyo.ac.jp

Sci. Adv. **12**, eaec7827 (2026)
DOI: 10.1126/sciadv.aec7827

The PDF file includes:

Figs. S1 to S7
Legends for data S1 and S2

Other Supplementary Material for this manuscript includes the following:

Data S1 and S2

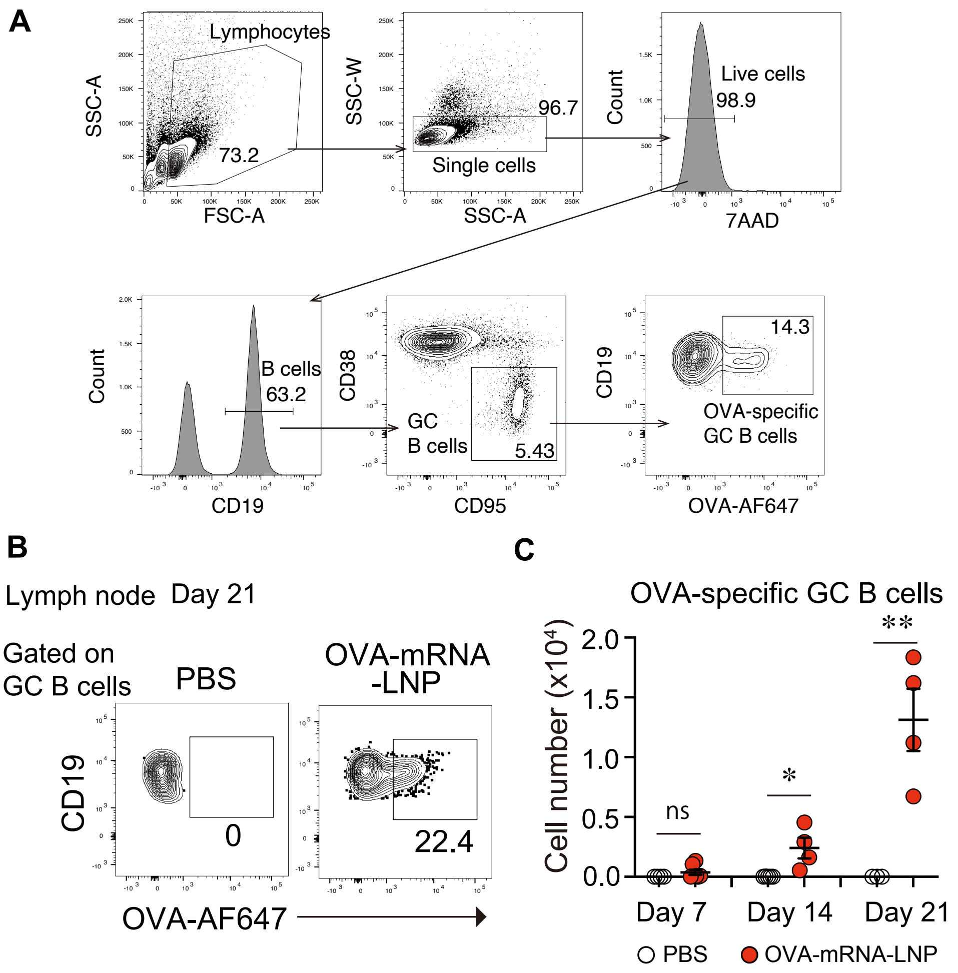

Figure S1. Detection of OVA-reactive germinal center B cells.

(**A**) Gating strategy for detection of OVA-reactive germinal center B cells.

(**B** and **C**) Representative flow cytometric analysis of OVA-reactive germinal center B cells in the lymph nodes on day 21. Graph shows the number of OVA-reactive germinal center B cells at each time point (n = 3–7 per group).

Statistical significance was determined by two-tailed Student's *t*-test for (B and C); *P < 0.05, **P < 0.01. ns, not significant.

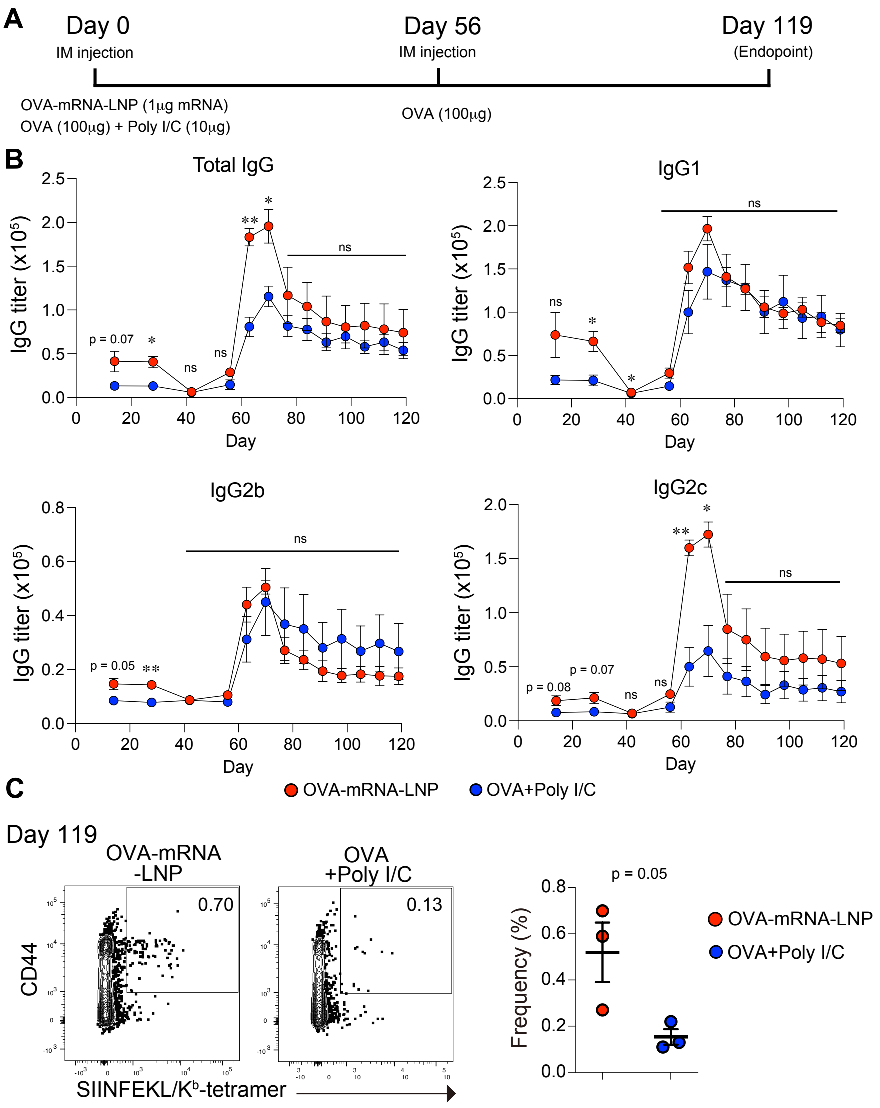

Figure S2. Durable immune responses induced by OVA mRNA-LNP.

(**A**) Experimental schedule of immunization. Mice were immunized intramuscularly with OVA mRNA-LNP or OVA protein (100 μg) + poly I/C on day 0 and boosted intramuscularly with OVA protein (100 μg) alone on day 56.

(**B**) Kinetics of serum OVA-specific IgG levels measured by ELISA (n = 3 mice per group).

(**C**) Flow cytometric analysis of OVA-specific CD8 T cells from the spleens of the indicated mice on day 119. The graph shows the frequency of OVA-specific CD8 T cells (n = 3 mice per group). Statistical significance was determined by two-tailed Student's *t*-test for (B and C); *P < 0.05, **P < 0.01. ns, not significant.

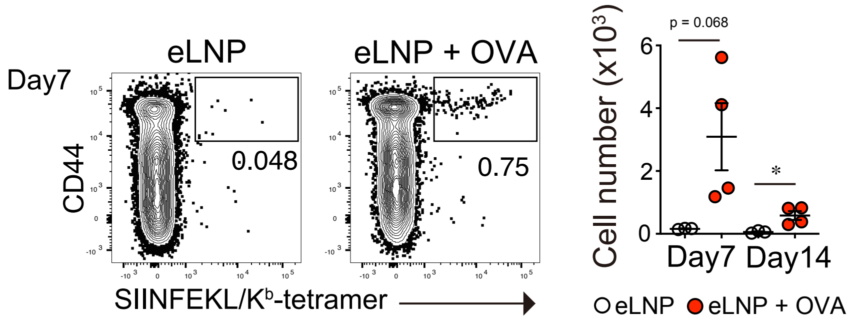

Figure S3. LNP acts as an adjuvant.

Flow cytometry analysis of OVA-specific CD8 T cells in the popliteal LNs of the indicated mice immunized with eLNP or eLNP + OVA protein. Representative plots are shown for day 7. The graph shows the cell number of OVA-specific CD8 T cells on days 7 and 14.

Statistical significance was determined by two-tailed Student's *t*-test; *P < 0.05.

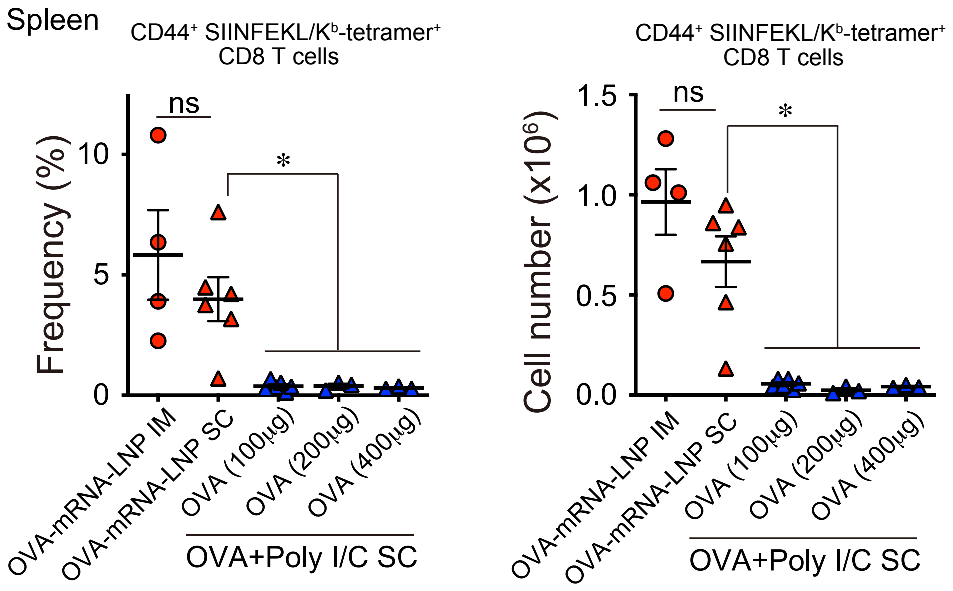

Figure S4. Comparison of administration routes for OVA mRNA-LNP.

OVA mRNA-LNP (1 μg RNA equivalent) was administered intramuscularly or subcutaneously, and the frequency and number of OVA-specific CD8 T cells in the spleen were analyzed 14 days later. In parallel, OVA protein (100, 200, or 400 μg) with poly I/C (10 μg) was administered subcutaneously, and the frequency and absolute number of OVA-reactive CD8 T cells in the spleen were evaluated.

Statistical significance was determined by one-way ANOVA followed by Dunnett's multiple comparisons test; * $P < 0.05$. ns, not significant.

A

Sample name	Cell number	Total reads	Unique reads
Vβ5⁺ EGFP⁺ CD8 T cells_1	363,090	291,135	16,600
Vβ5⁺ EGFP⁺ CD8 T cells_2	400,681	126,717	9,587
Vβ5⁺ EGFP⁺ CD8 T cells_3	401,394	167,407	9,769
OVA-mRNA-LNP tetramer⁺_1	43,766	144,920	2,134
OVA-mRNA-LNP tetramer⁺_2	50,220	136,331	2,152
OVA-mRNA-LNP tetramer⁺_3	32,638	130,054	2,360
OVA + PolyI/C tetramer⁺ _1	10,510	173,733	2,631
OVA + PolyI/C tetramer⁺ _2	36,981	225,056	2,035
OVA + PolyI/C tetramer⁺ _3	40,692	172,638	1,911

B

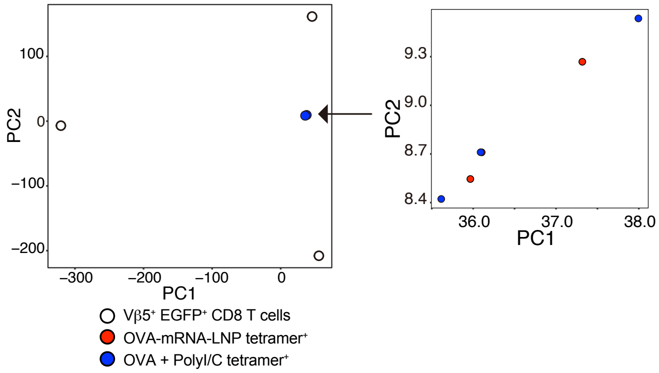

Figure S5. Summary of TCR repertoire sequencing.

(**A**) Vβ5⁺ EGFP⁺ CD8 T cells were purified from pre-immunized TCR-Vβ5-expressing retrogenic mice (n = 3). In parallel, GFP⁺ OVA-specific CD8 T cells were isolated from retrogenic mice immunized with either OVA-mRNA-LNP (n = 3) or OVA protein + poly I/C (n = 3). The number of input cells, total TCR reads, and unique TCR reads are shown.

(**B**) Principal component analysis of TCR frequency.

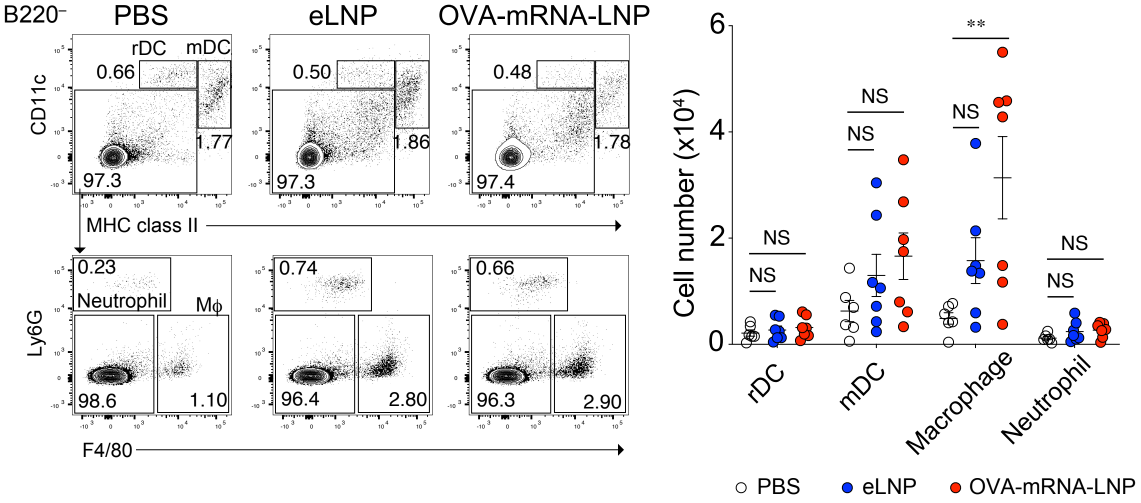

Figure S6. Antigen-presenting cells in dLNs following mRNA-LNP administration.

Mice were immunized with PBS, eLNP, or OVA-mRNA-LNP. One day later, popliteal LNs were collected and analyzed by flow cytometry.

(Upper) Representative plots showing CD11c and MHC class II expression in $B220^{-}$ cells.

(Lower) Representative plots showing Ly6G and F4/80 expression within the $B220^{-}$ $CD11c^{-}$ MHC class II^{-} population. The graph indicates the number of rDCs, mDCs, macrophages, and neutrophils. PBS, n = 6; eLNP, n = 7; OVA-mRNA-LNP, n = 7.

Statistical significance was determined by one-way ANOVA followed by Dunnett's multiple comparisons test; ** $P < 0.01$. ns, not significant.

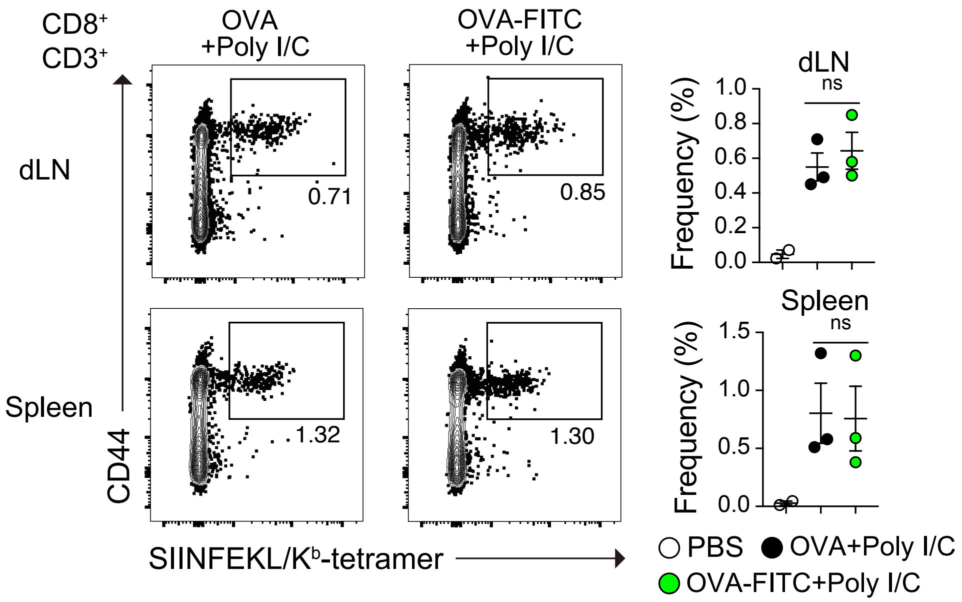

Figure S7. Normal differentiation of OVA-specific CD8 T cells by immunization with FITC-conjugated OVA protein.

Mice were intramuscularly injected with PBS, OVA protein (100 μg/mouse) + poly I/C, or FITC-conjugated OVA protein (100 μg/mouse) + poly I/C. Seven days later, OVA-specific CD8 T cells in the popliteal LNs and spleen were analyzed by flow cytometry. The graphs indicate the frequency of OVA-specific CD8 T cells in each tissue. PBS, n = 2; OVA + poly I/C n =3, OVA-FITC + poly I/C n =3. Statistical significance was determined by two-tailed Student's *t*-test. ns, not significant.

Auxiliary Supplementary Materials and Other Supporting Files

- **Raw data for TCR repertoire analysis.**

aec7827_Suppl. Other File Type_seq1_v1.csv

- **Frequency data for TCR clones used in PCA analysis, converted from the raw data.**

aec7827_Suppl. Other File Type_seq2_v1.csv
